# Closed-loop bioelectronic medicine for diabetes management

**DOI:** 10.1186/s42234-020-00046-4

**Published:** 2020-05-15

**Authors:** Amparo Güemes Gonzalez, Ralph Etienne-Cummings, Pantelis Georgiou

**Affiliations:** 1 Centre for Bio-Inspired Technology, Department of Electrical and Electronic Engineering, Imperial College London, London, UK; 2 Department of Electrical and Computer Engineering, Johns Hopkins University, Baltimore, USA

**Keywords:** Bioelectronic medicine, Closed-loop system, Diabetes, Vagus nerve, Neuromodulation

## Abstract

Modulation of the nervous system by delivering electrical or pharmaceutical agents has contributed to the development of novel treatments to serious health disorders. Recent advances in multidisciplinary research has enabled the emergence of a new powerful therapeutic approach called bioelectronic medicine. Bioelectronic medicine exploits the fact that every organ in our bodies is neurally innervated and thus electrical interfacing with peripheral nerves can be a potential pathway for diagnosing or treating diseases such as diabetes. In this context, a plethora of studies have confirmed the important role of the nervous system in maintaining a tight regulation of glucose homeostasis. This has initiated new research exploring the opportunities of bioelectronic medicine for improving glucose control in people with diabetes, including regulation of gastric emptying, insulin sensitivity, and secretion of pancreatic hormones. Moreover, the development of novel closed-loop strategies aims to provide effective, specific and safe interfacing with the nervous system, and thereby targeting the organ of interest. This is especially valuable in the context of chronic diseases such as diabetes, where closed-loop bioelectronic medicine promises to provide real-time, autonomous and patient-specific therapies. In this article, we present an overview of the state-of-the-art for closed-loop neuromodulation systems in relation to diabetes and discuss future related opportunities for management of this chronic disease.

## Background

A key biological process of every living system is the maintenance of homeostasis to ensure stability via continuous and rapid self-adjustments of the physiological state. The nervous system has a major role in preserving homeostasis using closed-loop mechanisms called neural reflexes. These include sensing, integration and effector circuitries that modulate the organ function. Interfacing with the neural reflexes may allow for a direct and efficient access to the rapidly changing internal conditions of the organism.

In recent decades, modulation of the nervous system through delivery of electrical or pharmaceutical agents has allowed the development of effective treatments to several severe clinical conditions (Shamji et al. 2017). Recently, we have seen the introduction of bioelectronic medicine, an evolution of neuromodulation, which aims to provide real-time and patient-specific therapies by modulating the activity of specific peripheral nerves to improve or restore impaired biological function of specific organs (Vitale and Litt 2018; Medicine A for AB 2020).

**Fig. 1:**
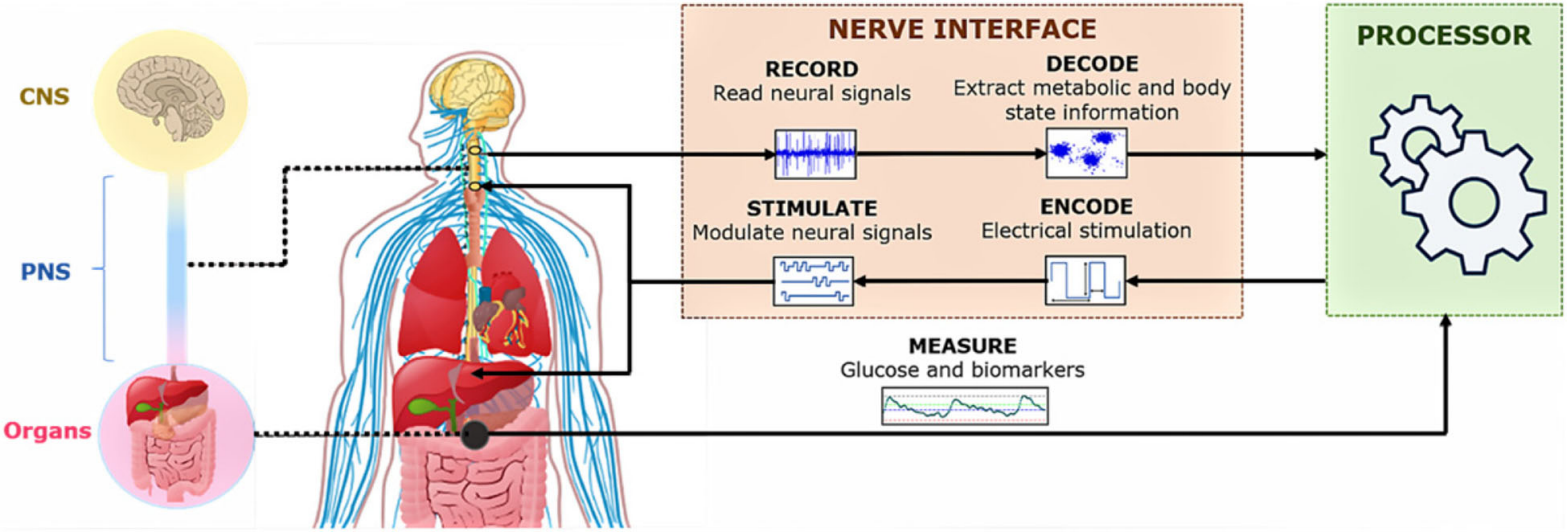
A depiction of future closed-loop neuromodulation systems for diabetes management. Metabolic biomarkers and neurophysiological recordings from a variety of peripheral nerves can be used to automatically control the stimulation dosage to be delivered back to the peripheral nervous system or directly to the organs to modulate their function

The therapeutic impact of bioelectronic medicine can be boosted by replicating the body’s closed-loop mechanisms: metabolic and neurophysiological biomarkers can be recorded and analyzed in real time to accordingly adjust the characteristics of the electrical stimulation delivered to the peripheral nerves or directly to the organs in order to modulate their function, as illustrated in Fig. 1 (Birmingham et al. 2014). Advances in bioelectronic medicine towards these closed-loop systems are supported by the development of new implantable technology and algorithms, which enable a safe, effective and minimally invasive interface with the nervous system (Rijnbeek et al. 2018; Zhou et al. 2018).

Initial research on bioelectronic medicine focused on the use of neuromodulation for treating inflammatory diseases by interfering with the neuro-immune reflex (Levine et al. 2014; Sundman and Olofsson 2014; Borovikova et al. 2000). Previous research confirmed that electrical stimulation of the vagus nerve modulates the production of inflammatory cytokines, and ultimately reduces the uncontrolled inflammatory activity associated with several disorders. This strategy has successfully improved the treatment of rheumatoid arthritis and colitis in clinical trials (Koopman et al. 2014), encouraging the application of bioelectronic medicine for the management of other chronic and severe diseases such as diabetes.

Diabetes is a chronic metabolic disease caused by an impairment of the hormone insulin which results in elevated blood glucose. This disorder currently affects 422 million people worldwide and is forecast to be the 7th leading cause of death in 2030 (World Health Organization, Organization WH 2016). Therapies involving insulin administration are indispensable for people with type 1 diabetes. For type 2, such therapies are sometimes used in early stages, and are critical at later stages of the disease. However, the costs associated with the insulin market are untenable, poised to reach $39.13 billion by 2020 based on the 2016 market research report published by MarketsandMarkets (MarketsandMarkets 2016). Moreover, the American Diabetes Association reported in March 2018 that the total costs of treating people diagnosed with diabetes has risen to $327 billion in 2017 from $245 billion in 2012 (American Diabetes Association AD 2018). These figures reflect the impact of diabetes on society and encourage the development of new treatments to improve the control of glucose. The application of bioelectronic medicine to treat diabetes has been previously criticized by some members of the scientific community due to the complexity of the underlying biological processes and the lack of legitimacy of preliminary simulations (Lowe 2016). However, recent work demonstrates the important role of the vagus nerve in the pathophysiology of diabetes and its comorbidities (e.g. cardiovascular diseases), and supports the potential benefits of modulating the peripheral nerve signals to improve the metabolic dysregulation (Steculorum et al. 2016; Zhang and van den Pol 2016).

### State of the art

The majority of research related to glucose control and neuromodulation has occurred in the context of metabolic disorders, specifically in the control of appetite and food intake in managing obesity (Aelen et al. 2008; Ruiz-Tovar et al. 2014; Ruiz-Tovar and Llavero 2016; Sohbati and Toumazou 2015; Johnson and Wilson 2018). Direct stimulation of the gastric myoelectrical activity with gastric pacemakers, known as gastric pacing, is one of the most exploited techniques (Greenway and Zheng 2007; Favretti et al. 2004; Cha 2014). For example, delivery of high frequency and short pulse width vagal stimulation in human clinical studies with the Transcend gastric pacemaker (Transneuronix, Mt. Arlington, NJ), inhibited the efferent vagal activity to the stomach (Greenway and Zheng 2007; Favretti et al. 2004; Cigaina 2004)., which caused an increased gastric distension and slowed gastric emptying. These changes led to earlier satiety, and eventually a reduction of food intake. The study reported a 20 to 40% of weight loss, depending on the patient. These findings on gastric pacing support further research on direct modulation of vagal activity known as vagal pacing. In relation to this, a human study in patients using vagus nerve stimulation to treat epilepsy reported a variety of responses, with slightly more than 50% of the patients losing between 5 and 10% of body weight (Burneo et al. 2002). More advanced strategies automatically modulate vagal activity in response to stomach distension and/or presence of satiating hormones, among other markers (Apovian et al. 2017; Shikora et al. 2015; Sarr et al. 2012; de Lartigue 2016; Yao et al. 2018; Cork 2018). A recent study in rats successfully reported up to a 38% body weight reduction by delivering biphasic electric pulses based on the peristalsis measured on the stomach (Yao et al. 2018).

Conversely, research on bioelectronic medicine for direct control of diabetes has emerged recently, with two main areas of interest: i) recording from the peripheral nerves to extract metabolic information and ii) modulating their electrical activity to improve glycaemic fluctuations. Regarding the former, recent work aims to use the vagus nerve as a glucometer to identify hypoglycaemic events from neural readings (Bedows 2018; Masi et al. 2019). In fact, neural activity presents a negatively correlated response to glycemia (Masi et al. 2019). The complexity of the recorded data requires advanced strategies to identify and decode neural signals related to glycaemic levels. In particular, Masi et al. (2019) have developed a decoding algorithm that recreates blood glucose levels with high accuracy using regression models with regularization to avoid over-fitting (Masi et al. 2019). Preliminary results are promising and encourage application of their method for glucose monitoring and control in the near future, especially for type 1 diabetes.

Selective electrical activation or blockade of specific vagal fibers to specific organs has been shown to be a way to impact different metabolic processes resulting in different glycaemic outcomes. Vagal blocking of afferent/efferent fibers has been experimentally achieved by direct transection of the vagus nerve (Meyers et al. 2016), however this methodology cannot be translated into clinical research in humans. As a result, ongoing research is focused on finding a combination of parameters that can selectively block specific fiber types, or the afferent/efferent traffic (Johnson and Wilson 2018). One such technique uses high frequency stimulation targeting a variety of nerves and locations. For example, this strategy has shown to successfully block nerves conveying pain signals, making it an alternative efficient method to conventional spinal cord stimulation in the treatment of chronic pain (Kumar et al. 2018). As another example, Shikora et al. used intermittent vagal blocking at the level of the abdomen in people with type 2 diabetes, and reported a reduction in body weight and improvement of glycaemic control based on enhanced levels of HbA1c, which is a marker for diabetes development (Shikora et al. 2013). Further research has targeted the activity of the carotid sinus nerve (Sacramento et al. 2016; Sacramento et al. 2018; Conde and Guarino 2018). In this domain, Conde et al. successfully restored insulin sensitivity and glucose tolerance in people with type 2 diabetes by blocking the activity of the carotid sinus nerve using kilohertz frequency alternating current (KHFAC) modulation (Sacramento et al. 2018; Conde and Guarino 2018).

The impact of neurostimulation on type 1 diabetes has been less explored, but recent research from Guyot et al. (2019) suggests that stimulation of the pancreatic sympathetic nerves projecting to the pancreatic lymph nodes inhibits the progression of the disease, at least in mice (Guyot et al. 2019). Activation of these nerves with a stimulation frequency of 10 Hz and 450 μA amplitude resulted in a reduction of pro-inflammatory cytokines and of the proliferation of autoreactive T cells, therefore limiting the progression of the disease. Preliminary optimization of the stimulation parameters eliminated undesired effects such as blood flow alteration and axonal excitability exhaustion, hence allowing for therapeutic use.

It is also possible to directly stimulate the organs of interest to regulate their function. For example, acute electrical stimulation of the parenchyma tissue of the liver for 90 min resulted in a reduction of both fasting and postprandial glucose levels in the healthy state and type 1 and type 2 diabetic rats (Chen et al. 2010). Moreover, continuous stimulation for 8 h during 4 consecutive days not only resulted in decreased glucose levels in the three groups, but also delayed gastric empty and increased plasma glucagon-like peptide-1 (GLP-1) level. Adverse effects were not reported in either case, making it a feasible and safe approach for glycaemic control.

Finally, inflammation has been found to be related to obesity-induced insulin resistance in people with type 2 diabetes (Esser et al. 2015), as inflammatory cytokines such as TNF can directly induce insulin resistance (Chang et al. 2019; GKS et al. 1996). In addition, reduced vagal activity has been reported in these patients (Chang et al. 2019). As a result, modulation of the immune reflex arc using vagal activation might provide pathway to alleviate both the inflammatory and the metabolic dysregulation of these patients (Esser et al. 2015; Chang et al. 2019).

### Future directions

The studies cited above are examples of current applications of bioelectronic medicine in diabetes. However, there is a plethora of opportunity to target the processes and organs involved in the glucose-response reflex through modulation of the nervous system (Güemes and Georgiou 2018). To begin with, recent research has given insight into neural control of the liver and its implication on the balance of hepatic glucose production and uptake (Yi et al. 2010; Van Den Hoek et al. 2008; Kalsbeek et al. 2004; Matsuhisa et al. 2000). As a result, modulating the neural signals to the liver would reduce the risk of hypoglycaemic events, especially for type 1 diabetes, by enhancing endogenous glucose production, which would rapidly increase glucose levels.

Secretion of hormones from the pancreas is also under the control of the nervous system (Meyers et al. 2016; Thorens 2011; Ahrén 2000; Ahren et al. 1987). These neural pathways could be modulated to regulate the secretion of insulin and glucagon in patients with type 2 diabetes and early stages of type 1 diabetes, where there are still a considerable number of β-cells intact. This can be particularly desirable in situations where glucose control in diabetic subjects is challenging, such as during exercise, which results in a decrease in glucose levels. Prompt inhibition of insulin secretion and a promotion of counterregulatory hormone secretion (such as glucagon) before starting exercise may also be used to improve control of glucose fluctuations and reduce the risk of hypoglycaemia.

Neuromodulation of pancreatic secretion could be further applied in patients with stem cell-derived β-cell transplants. This strategy has been found to improve glycaemic control in pre-clinical trials, but many challenges are yet to be resolved, including restoration of vascularization and neural innervation. Successful advances are being made in the former, but full restoration of the functionality of the transplanted islets is still far from optimal. To address this issue, Seicol et al. (2019) propose the development of biocircuit-augmented islet transplants containing mature, vascularized and neural innervated β-cells, where neurons will grow inside hydrogel-based micro tissue engineered neural network (micro-TENN) scaffolds (Seicol et al. 2019). We proposed bringing this idea one step further and using closed-loop neuromodulation of neural bio-circuits to restore the physiologic response to neural signalling that is present in healthy individuals (Güemes and Georgiou 2018).

Control of insulin sensitivity through neuromodulation, although generally relevant to type 2 diabetes, could be further extended for use in people with type 1 diabetes. These subjects require external injection of the insulin to lower glucose levels after a meal intake. Modulation of the neural pathways could increase the effect of this exogenous insulin in lowering blood glucose (i.e. insulin sensitivity) (Güemes et al. 2019).

The opportunities of closed-loop neuromodulation should not be restricted to treatment of early and late stage diabetes. Vagus nerve recordings, as previously demonstrated (Bedows 2018), can also be used as a monitoring tool of the glycaemic state for early detection of the disease and to control its progression (Medicine A for AB 2020).

Finally, it is crucial to consider the integration of this new strategy for glucose control with the systems that are currently implemented for diabetes management. In particular, closed-loop systems for insulin delivery, such as the artificial pancreas, are regarded as cutting-edge technology in glucose management for diabetes (Ramli et al. 2019; Taleb et al. 2019; Kovatchev 2018; Saunders et al. 2019; Herrero et al. 2019). An artificial pancreas comprises a sensor for continuous glucose monitoring, an insulin pump and an algorithm that replicates the endocrine function of a healthy pancreas and calculates the optimal dose to be delivered based on the real-time glucose readings from the sensor. One of the most employed artificial pancreas systems is the Medtronic 670G (Medtronic, Northridge, CA, USA), which is approved by the US Food and Drug for use in people with type 1 diabetes over 14 years of age (PMA P130013: FDA Summary of Safety and Effectiveness Data 2013), and is also CE Mark approved for use in people over 7 years of age within Europe. Despite the improved glycaemic control recently reported in clinical trials (Brown et al. 2019; Bailey et al. 2019), the system is still not fully autonomous and requires user input, especially during meal intake (Ramli et al. 2019; Gingras et al. 2018). Incorporating the opportunities that bioelectronic medicine offers for diabetes will undoubtedly enable improved computation of the insulin doses, increased autonomy, and personalized glucose control.

### Challenges

In this article, current and future opportunities for applying bioelectronic medicine for improving diabetes have been presented, all of which would further benefit from the incorporation of a “closed-loop” control of both the immune and metabolic reflexes. That said, some important challenges and advances must be addressed in order to translate these strategies into real closed-loop therapeutic approaches.

Selective activation of the specific neural fibers that target the biological processes of interest is a major requirement to minimize the risk of undesired activation of other tissues, organs, and processes. This entails the development of novel stimulation strategies and devices to allow access and modulation of the activity of individual fibers. Moreover, stimulation selectivity relies on having a clear picture of the anatomy of the peripheral nervous system, and of the role of the individual fibers in healthy and disease conditions. This challenge is related with the issue of transferability, not only between species, but also between nerves and tissues in the same species. This challenge of transferability, not only between species but also between nerves and tissues in the same species, arises from the anatomical and conduction differences among nerve fibers in the peripheral nervous system, which can affect their sensitivity and response to electrical stimulation (Günter et al. 2019). For example, the pancreatic branch differs from the gastric-projecting neurons in that they have i) longer duration of action potentials, ii) longer after-hyperpolarization decay time, iii) smaller soma area and iv) larger diameter (Babic and Travagli 2016). More research is needed to overcome this limitation, as only hypothetical extrapolation of the results is possible due to a lack of studies providing a comprehensive view of the similarities/divergences of parameters between nerve fibers and species.

It is also necessary to acquire a better understanding of the electrode-nerve interface and the design of new electrode materials and fabrication strategies that ensure safety during stimulation, reliability during chronic recording, full biostability and biocompatibility with the surrounding tissue, and long term preservation of their functionality (Günter et al. 2019; Walker et al. 2009; Luan et al. 2014; Zanos 2019)*.*

Improved neural decoding algorithms, coupled with advances in data analysis techniques, are allowing us to extract real-time information about the state of the nerve and the related organs (Vitale and Litt 2018; Zanos 2019). Combining the processing of these neural recordings with data acquired during continuous monitoring of other biomarkers, and capitalizing on machine learning algorithms, presents an ideal strategy to characterize the state of the biological processes of interest in great detail.

Current artificial pancreas systems also have several challenges that need to be addressed if they are to be used in conjunction with bioelectronic medicine. Among them, the delays in interstitial glucose sensing and hormonal subcutaneous absorption (Ramli et al. 2019; Taleb et al. 2019; Gingras et al. 2018; Herrero et al. 2017), failures in the insulin pump (Meneghetti et al. 2018), and glycaemic control during meals (Ramkissoon et al. 2018) and exercise (Ramkissoon et al. 2020) are the most critical. Despite these challenges, the use of the artificial pancreas is associated with high levels of satisfaction and quality of life, and reduced fear of hypoglycaemic events among the users (Ramli et al. 2019). Moreover, there are ongoing strategies for educating the users to understand and operate these closed-loop systems (Lewis 2018; Ehrmann et al. 2018; Smeets et al. 2010). These observations suggest that novel technology integrating closed-loop neuromodulation and metabolic systems will be efficiently implemented and successfully adopted by the diabetes community.

A final challenge involves integrating all the recordings and acquired information into patient-specific closed-loop systems that adaptively adjust stimulation parameters to selectively drive nerve fiber functionality to achieve the desired state that restores or maintains homeostasis. To achieve this ultimate goal, innovative solutions for data transmission and storage, and power supply, are needed.

## Conclusion

Closed-loop neuromodulation of metabolic dysfunction is a promising new pathway to treat diabetes, as conceptualized in Fig. 1. To fully exploit the potential benefits of bioelectronic medicine as a new form of therapy, however, medicine and technology should work in synchrony. Advances in minimally invasive technology to reliably interface with the nervous system are needed to take us one step closer to using bioelectronic therapies for optimal metabolic control in daily life. Accordingly, it is crucial to fully understand the biological processes that underlie the glucose-response reflex to enable the creation of more efficient and safe systems. We believe that 1 day this will become a reality and provide personalized diagnostics and treatment for people with diabetes.

## Data Availability

Not applicable.

## Abbreviations

GLP-1: Glucagon-like peptide-1
KHFAC: Kilohertz frequency alternating current
TENN: Tissue-engineered neural network
